# Effect of *Flos carthami* Extract and *α*_1_-Adrenergic Antagonists on the Porcine Proximal Ureteral Peristalsis

**DOI:** 10.1155/2014/437803

**Published:** 2014-07-09

**Authors:** San-Yuan Wu, Kee-Ming Man, Jui-Lung Shen, Huey-Yi Chen, Chiao-Hui Chang, Fuu-Jen Tsai, Wen-Tsong Hsieh, Daniel Winardi, Yuan-Ju Lee, Kao-Sung Tsai, Yu-Ning Lin, Yung-Hsiang Chen, Wen-Chi Chen

**Affiliations:** ^1^Department of Anesthesiology, Tungs' Taichung MetroHarbor Hospital, Taichung 435, Taiwan; ^2^Graduate Institute of Integrated Medicine, Institute of Chinese Medicine, School of Chinese Medicine, Department of Pharmacology, Research Center for Chinese Medicine and Acupuncture, China Medical University, Taichung 404, Taiwan; ^3^Department of Medicinal Botanicals and Health Applications, Da-Yeh University, Changhua 515, Taiwan; ^4^Department of Life Sciences, National Chung Hsing University, Taichung 402, Taiwan; ^5^Graduate Institute of Geriatric Medicine, Anhui Medical University, Hefei 230032, China; ^6^Department of Dermatology, Taichung Veterans General Hospital, Taichung 407, Taiwan; ^7^Departments of Obstetrics and Gynecology, Medical Genetics and Pediatrics, Dermatology, Medical Research, and Urology, China Medical University Hospital, Taichung 404, Taiwan; ^8^Department of Urology, National Taiwan University Hospital, Taipei 100, Taiwan

## Abstract

Traditional Chinese medicine (TCM) has been proposed to prevent urolithiasis. In China,* Flos carthami* (FC, also known as* Carthamus tinctorius*) (Safflower; Chinese name: Hong Hua/紅花) has been used to treat urological diseases for centuries. We previously performed a screening and confirmed the* in vivo* antilithic effect of FC extract. Here,* ex vivo* organ bath experiment was further performed to study the effect of FC extract on the inhibition of phenylepinephrine (PE) (10^−4^ and 10^−3^ M) ureteral peristalsis of porcine ureters with several *α*_1_-adrenergic antagonists (doxazosin, tamsulosin, and terazosin) as experimental controls. The results showed that doxazosin, tamsulosin, and terazosin dose (approximately 4.5 × 10^−6^ − 4.5 × 10^−1^ *μ*g/mL) dependently inhibited both 10^−4^ and 10^−3^ M PE-induced ureteral peristalsis. FC extract achieved 6.2% ± 10.1%, 21.8% ± 6.8%, and 24.0% ± 5.6% inhibitions of 10^−4^ M PE-induced peristalsis at doses of 5 × 10^3^, 1 × 10^4^, and 2 × 10^4^ *μ*g/mL, respectively, since FC extract was unable to completely inhibit PE-induced ureteral peristalsis, suggesting the antilithic effect of FC extract is related to mechanisms other than modulation of ureteral peristalsis.

## 1. Introduction

Ureteral stones can obstruct the ureter and cause severe colicky pain as a sequence of hydronephrosis and increased intrarenal pressure [1–3]. The clinical symptoms of renal colic include acute colicky pain, urinary frequency, dysuria, nausea, and vomiting. As the stone migrates downward from the kidney or upper ureter to the distal ureter, treatment involves measures to relieve pain, encouraging water intake, and administering some medications [4, 5]. Spontaneous expulsion is expected within 4 weeks if the stone is <6 mm in diameter [6]. For situations other than those requiring urgent treatment, such as larger stone size, infection, intractable pain, renal function deterioration, or solitary kidney, many treatments are available to induce spontaneous stone passage.

Drugs commonly used to treat colic include nonsteroidal anti-inflammatory drugs (NSAIDs), narcotics, antidiuretics, calcium channel blockers, and *α*-blockers. Several clinical trials of highly selective *α*-blockers are promising for improving spontaneous ureteral stone expulsion [7]. However, the efficacy of these treatments remains controversial. Studies by Wang et al. found that tamsulosin improved the success rate of medical expulsion therapy (MET) of stones in selected patients [8]. A systematic review by Singh et al. recommended the use of *α*-antagonists or calcium channel blockers to facilitate ureteral stone expulsion [9]. A meta-analysis by Woolley concluded that tamsulosin may be useful for enhancing ureteric stone expulsion [10]. However, tamsulosin treatment did not demonstrate improvements in the rate of stone expulsion in patients with distal ureteral stones that are ≤7 mm in diameter. Tamsulosin treatment was shown to have a supportive analgesic effect in a randomised, double-blind, placebo-controlled trial by Hermanns et al. [11]. A study of Mexican patients by Ochoa-Gómez et al. did not find that tamsulosin had greater efficacy than conventional treatment [12]. Nevertheless, the effect of other nonselective *α*-blockers on MET remains to be determined.

The effect of *α*-blockers on MET is mediated through ureteral peristalsis. The ureter has differential distribution of *α*-adrenergic receptors. Blocking receptor action may relax the ureteral peristalsis that is believed to facilitate stone expulsion. Davenport et al. studied the relaxation effect of selective *α*_1A_-adrenergic antagonists on human ureteral activity [13]. Hernandez et al. investigated ureteral peristalsis in porcine ureter and described some useful methods [14]. In this study, we used porcine ureteral smooth muscle samples to study the effect of some potential drugs useful for MET.

Traditional Chinese Medicine (TCM) [15–17] has been proposed to play a role in the prevention of urolithiasis [18].* Flos carthami* (FC, also known as* Carthamus tinctorius*) (Safflower; Chinese name: Hong Hua/紅花) has been used to treat urological diseases for centuries in China [19]. We previously confirmed the* in vivo* antilithic effect of FC extract [20]; here, we assessed the possible effect of FC extract on the inhibition of ureteral peristalsis. *α*-adrenergic receptors are found on arteries and in the urinary tract. However, few animal [21] and clinical studies have assessed the effect of FC extract on ureter. The purpose of this study was to investigate the inhibitory effect of FC extract on peristalsis in an* ex vivo* porcine ureteral model in organ bath experiment.

## 2. Materials and Methods

### 2.1. Porcine Ureter

Porcine ureters from healthy animals were kindly provided by the slaughterhouse in Taichung City. The ureteral samples were immediately put into a flask of preoxygenated Krebs' solution (pH 7.4) at 4°C after the pigs were slaughtered in the house.

### 2.2. Ureteral Peristalsis Measurement

**Figure 1:**
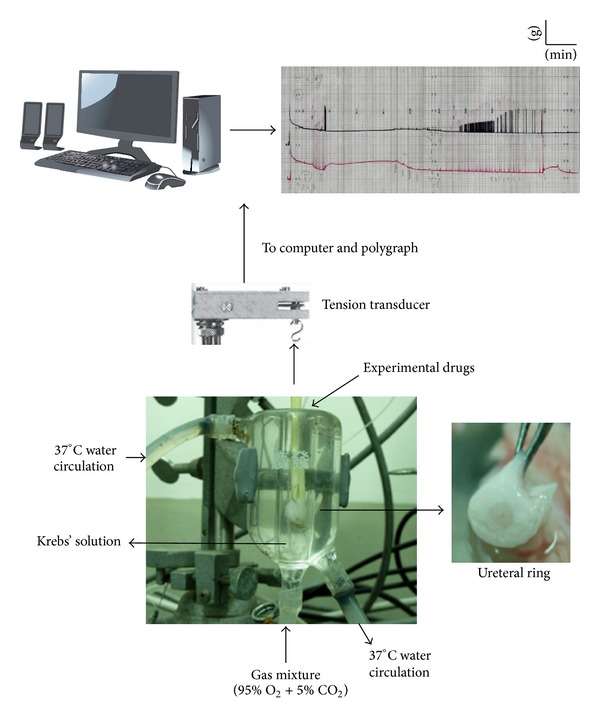
A schematic diagram of a porcine ureteral ring preparation in an organ bath.

The method for measuring ureteral contraction was previously described by Hernandez et al. with modifications (Figure 1) [14, 22]. Briefly, the ureteral samples were cut longitudinally into 1 cm strips. The system used open-ended Perspex tissue baths with a volume of 5 mL to allow exchange with physiological solution.

Tension transducers (Gould 2600 polygraph, Gould Instruments, Cleveland, OH, USA) were used to convert the mechanical tension of the sample into a voltage signal. The tension transducer is suspended by a retort stand vertically above the tissue bath [23], and fine cotton sutures attach the muscle specimen to the transducer; the opposite end of the sample was fixed within the tissue bath. The specimens were continuously irrigated with Krebs' solution with gas (95% oxygen + 5% carbon dioxide). Nonporous tubing connected the Krebs' solution to the tissue baths via a peristaltic pump delivering 1 mL/min to each tissue bath (Minipuls 2, Gilson Inc., Middleton, WI, USA). The tubing passed through a water bath set at 37°C to ensure that the perfusate was at body temperature by the time it reached the tissue bath. The tension transducer responses were recorded on polygraph paper.

We simultaneously assessed cut ureteral samples in each experiment. The ureteral rings were irrigated with Krebs' solution for 1 h to allow equilibration, during that time 5 g of tension was applied. Any spontaneous activity was recorded. Viability was determined by applying 80 mM potassium-enriched Krebs' solution to produce near-maximal contraction. Potassium-enriched solutions were prepared by substituting potassium for sodium [24]. Any unresponsive ureteral samples (unchanged on polygraph during baseline tension) were discarded.

The tension generated with 80 mM potassium-enriched Krebs' solution was used as the control value. The drugs were added to 80 mM potassium-enriched Krebs' solution in increasing concentrations and applied consecutively with a 10 min washout with Krebs' solution between each concentration. Phenylepinephrine (PE) was used to induce ureteral peristalsis at concentrations of 10^−4^ M and 10^−3^ M. The inhibitory effects of drugs were compared with the effects elicited by PE.

### 2.3. Drug Preparation

Only one drug was applied to each ureteral sample to prevent any risk of cross-reactivity. The maximum tone achieved at 3 min was recorded and expressed as a percentage of that recorded with potassium-enriched solution alone. Before testing the drug solutions, 80 mM potassium solution (prepared in double-distilled (dd) water) was applied to ureteral specimens. The maximum tone generated did not increase on repeated application of 80 mM potassium solution.

The following drugs were tested: water extracts of FC, doxazosin, terazosin, (two nonselective *α*_1A_-adrenoceptor antagonists), and tamsulosin (selective *α*_1A_-adrenoceptor antagonist). The tested concentrations of all *α*-blockers were 10^−10^ M, 10^−9^, 10^−8^, 10^−7^, and 10^−6^ M (approximately equal to 4.5 × 10^−6^–4.5 × 10^−1^ *μ*g/mL) dissolved in dd water. All chemical agents were obtained from Sigma-Aldrich Inc. (St. Louis, MO, USA).

We used herbal powder of FC extract provided by the Koda pharmaceutical company (Taoyun, Taiwan). FC powder quality including high-performance liquid chromatography finger print, thin layer chromatography, and heavy metal quantification was examined by the Medical and Pharmaceutical Industry Technology and Development Centre (Taiwan) [20]. The highest stock dose of FC extract was the maximal dissolved dose in dd water (2.0 g/mL water), and the low and medium doses were 0.5 and 1.0 g/mL, respectively. For each experiment, 50 *μ*L was added to the 5 mL organ bath (final concentration: 5 × 10^3^, 1 × 10^4^, and 2 × 10^4^ *μ*g/mL, resp.).

The inhibitory effect on peristalsis frequency was determined by comparing the values after treatment with those observed at baseline. The percentage of inhibition was calculated by comparing the frequency from baseline to the induction period (3 min interval). Each test was repeated in quadruplicate, and the results are expressed as mean ± standard error of the mean (SEM).

Percentage of inhibition = (baseline frequency − induction frequency)/baseline frequency × 100%. The concentration of 50% inhibition (IC_50_) was calculated to compare the doses of tested agents. IC_50_ was calculated with a linear curve and the equation for a line is (*Y* = *aX* + *b*), where “*a*” and “*b*” are the constants of the equation, “*Y*” is the inhibition of peristalsis (%), and “*X*” is the drug concentration (*μ*g/mL).

### 2.4. Statistical Analyses

The significance of differences in mean values among groups was determined with analysis of variance. The data are expressed as means ± SEM. Differences were considered statistically significant if *P* < 0.05. All analyses were performed with the Statistical Package for the Social Science (SPSS for Windows, release 15.0, SPSS Inc., Chicago, IL, USA).

## 3. Results

### 3.1. Effects of Nonselective *α*-Blockers: Doxazosin, Tamsulosin, and Terazosin

**Figure 2:**
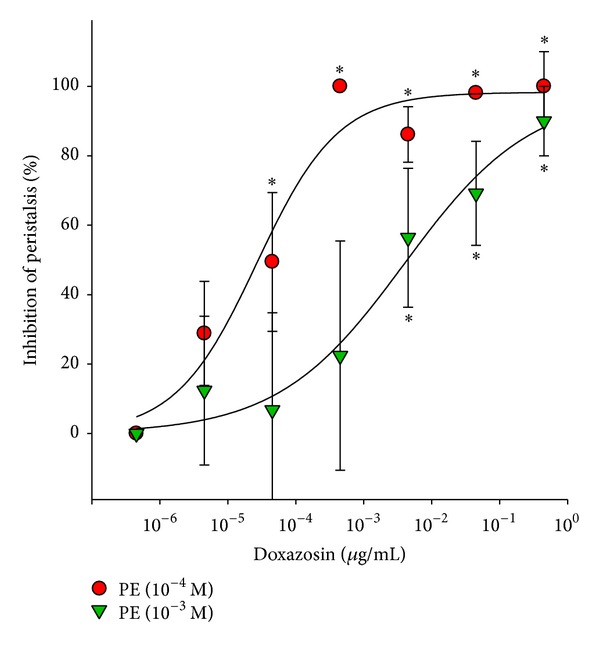
Inhibitory effect of doxazosin on porcine ureteral peristalsis. Graphic representation of concentration-response curves. The calculated data were presented as mean ± SEM for at least four different experiments. *P < 0.05 compared to control group.

**Figure 3:**
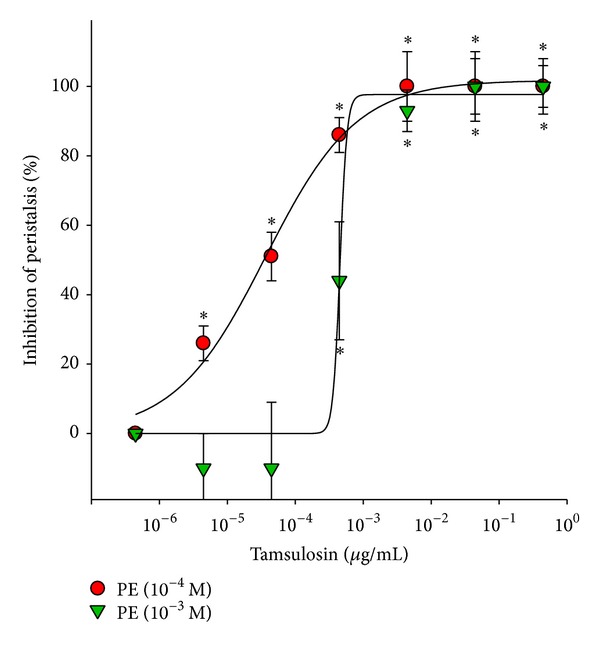
Inhibitory effect of tamsulosin on porcine ureteral peristalsis. Graphic representation of concentration-response curves. The calculated data were presented as mean ± SEM for at least four different experiments. *P < 0.05 compared to control group.

**Figure 4:**
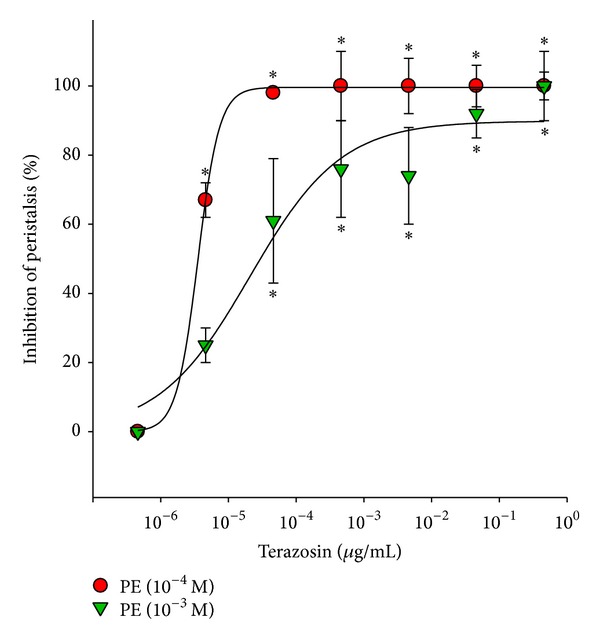
Inhibitory effect of terazosin on porcine ureteral peristalsis. Graphic representation of concentration-response curves. The calculated data were presented as mean ± SEM for at least four different experiments. *P < 0.05 compared to control group.

A total of 100 ureteral samples were used in this study, and 88 were viable for the experiment. Ureteral peristalsis was recorded on polygraph paper. All tested *α*_1_-adrenoceptor antagonists exerted an inhibitory effect on PE-induced ureteral peristalsis. Doxazosin (Figure 2), tamsulosin (Figure 3), and terazosin (Figure 4) dose dependently inhibited both 10^−4^ and 10^−3^ M PE-induced ureteral peristalsis.

### 3.2. Effects of FC Extract

**Figure 5:**
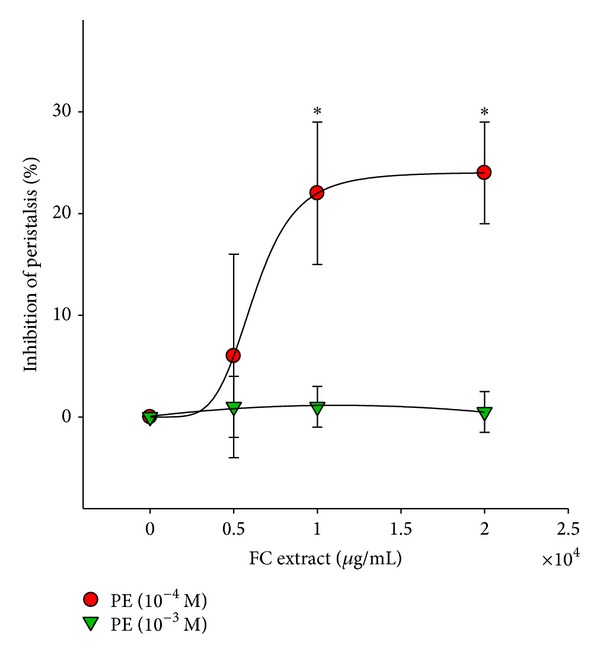
Effect of FC extract on porcine ureteral peristalsis. Graphic representation of concentration-response curves. The calculated data were presented as mean ± SEM for at least four different experiments. *P < 0.05 compared to control group.

FC-mediated inhibition was observed in 10^−4^ M PE-treated samples, and the levels of inhibition were 6.2 ± 10.1%, 21.8 ± 6.8%, and 24.0 ± 5.6% for the doses of 5 × 10^3^, 1 × 10^4^, and 2 × 10^4^ *μ*g/mL, respectively. However, FC extract did not have an inhibitory effect on 10^−3^ M PE-induced ureteral peristalsis (Figure 5). The IC_50_ of tested agents on the porcine proximal ureteral peristalsis is shown in Table 1.

## 4. Discussion

The results indicate that the tested agents inhibited PE-induced ureteral peristalsis in isolated porcine proximal ureter. Terazosin and tamsulosin had greater inhibitory effects than doxazosin. By contrast, FC extract only showed a weak inhibitory effect at concentrations of PE (10^−4^ M), with up to 24.0% inhibition at the highest dose. FC extract was ineffective at the higher concentration of PE (10^−3^ M)-induced ureteral peristalsis.

We used three *α*-blockers as experimental controls, and all of them exerted dose-dependent inhibitory effects on ureteral peristalsis. There are numerous studies describing the inhibitory effect that *α*-blockers have on distal ureteral peristalsis. Although there is a lack of innervation of the ureter, stimulation of *α*_1_ adrenergic receptors may enhance its contraction. Therefore, *α*-blockers are hypothesised to have pharmacological effects that facilitate ureteral stone passage. Currently, *α*-blockers such as tamsulosin, terazosin, and doxazosin are used to treat lower urinary tract symptoms, and several clinical trials have suggested that these agents are helpful for MET. A randomised clinical trial by Červenàkov et al. indicated that tamsulosin increases stone expulsion rate [25].

Zaytoun et al. also studied the effects of tamsulosin and doxazosin as adjunctive therapies following shock wave treatment for renal stones in a randomised controlled clinical trial [26]. They found that both *α*-blockers decreased the time of stone expulsion, the amount of analgesic use, and the number of colics but did not find increased rates of stone expulsion. Tamsulosin more effectively decreased the number of colic episodes and decreased analgesic doses compared to doxazosin. This trial lasted up to 12 weeks and enrolled patients with renal stones <2 cm; they did not specifically assess ureteral stones, and it is possible that the *α*-blockers affected the entire ureter. MET has been studied extensively in recent years. Most reports confirmed that *α*-blockers facilitate stone passage spontaneously without shock wave treatment, especially in the distal ureter.

We investigated the effects of FC extract and *α*-blockers on proximal porcine ureter, which is less studied than the distal segment. Only a few reports have measured autonomic receptors in ureteral smooth muscle. Furthermore, it is difficult to maintain stable spontaneous contractions in the ureter, and either electrical field stimulation or high concentrations of KCl are utilized to induce ureteral contractions in many* in vitro* ureteral pharmacological examinations. We avoided these confounding variables by employing spiral ureteral strips, which generate spontaneous contractions.

The existence of *α*_1_-, *α*_2_-, and *β*-adrenoceptors and muscarinic cholinergic receptors were demonstrated in the canine ureter using radio-ligand techniques. The density of *α*_1_-receptor-binding sites was significantly greater than that of other receptors examined. Morita et al. showed that the sympathetic nervous system is more involved than the parasympathetic nervous system in canine ureteral contractile activities and that *α*- and *β*-receptors in canine ureteral smooth muscle are comprised mainly of the *α*_1_- and *β*-subtypes. Their results also suggested that prostaglandins directly affect canine ureteral contraction [27].

There are several reports on the use of TCM in the management of urinary stone disease [18]; the pharmacological effects include increased urinary volume, decreased crystal formation, and decreased secretion of promoter [28]. Purportedly, TCM treatments have fewer side effects than other antilithic treatments. We previously studied FC extract in a rat model and found a positive effect on the prevention of stone occurrence. However, delayed blood coagulation was also found [20]. This indicated that a large dose of single agent was inappropriate for the treatment of stone disease. There were fewer studies on the inhibition of ureteral peristalsis for MET; the present study of a TCM compound on the treatment of MET indicated a positive effect.

FC extract exerted inhibitory effects on the peristalsis of porcine proximal ureter, especially at the highest dose. Because FC extract was difficult to dissolve, further study is needed to achieve a dose that would match the effects achieved by *α*-blockers. Traditionally, a single herbal drug is not used to treat disease [29–31]; rather, most treatments are formulas. In addition, our previous work indicated that FC extract also affects blood coagulation [32]. For these reasons, increased dose of FC extract might be inappropriate for the treatment of stone disease. However, we believe that a combination of FC extract with another herbal medicine might be effective for MET.

This study has inherent advantages and limitations. First, the porcine ureter was useful in that we were able to obtain a large amount of viable samples. The experimental period time was short, and the results were easily observed and calculated. However, pig ureters may be not an ideal model for humans. For example, the variation (SEM) is so high in lower concentration of both data of *α*-adrenergic antagonists and FC extract. It may be caused because of the variances of the individual animal tissues. Second, the concentration of FC extract was limited, and we were unable to induce an inhibitory effect that was comparable with those achieved with *α*-blockers. Page et al. studied the effects of oral alfuzosin on ureteral pressure and peristalsis in a distally obstructed porcine ureter. They showed the increase in ureteral pressure and peristaltic rate with distal ureteral obstruction. Alfuzosin appears to decrease the delta pressure in the distal ureter during obstruction; even statistical significance was not reached [33]. The opposite might be because of the difference between* in vivo* porcine study with oral administration of alfuzosin and* ex vivo* organ bath study. Further assessment of FC extract on MET should include oral administration in an animal model to increase the available dose. Moreover, a number of components in FC extract have been isolated, such as safflor yellow and carthamin [34]. Further studies on these effective constituents using this approach would be beneficiary.

## 5. Conclusions

In conclusion, all the tested *α*-blockers exerted inhibitory effects on porcine ureteral peristalsis in a dose-dependent manner. However, FC extract did not achieve the same inhibitory effect as the *α*-blockers. The present work assessed the effect of FC extract on ureter, which might provide clue for future mechanism of TCM studies. Since FC extract was unable to match the effects of *α*-blockers on inhibiting PE-induced ureteral peristalsis, suggesting the antilithic effect of FC extract is related to mechanisms other than modulation of ureteral peristalsis.

## Figures and Tables

**Table 1:** The concentration of 50% inhibition (IC_50_) of tested agents on the porcine proximal ureteral peristalsis.

IC_50_ (*μ*g/mL)	Doxazosin	Tamsulosin	Terazosin	FC extract
PE (10^−4^ M)	2.9 × 10^−5^	3.7 × 10^−5^	3.9 × 10^−6^	—
PE (10^−3^ M)	5.1 × 10^−3^	4.9 × 10^−4^	3.2 × 10^−5^	—

FC: *Flos carthami*.

PE: phenylepinephrine.
